# Preparation of graphene nanocomposites from aqueous silver nitrate using graphene oxide’s peroxidase-like and carbocatalytic properties

**DOI:** 10.1038/s41598-020-61929-9

**Published:** 2020-03-20

**Authors:** Kunal Garg, Petri Papponen, Andreas Johansson, Nitipon Puttaraksa, Leona Gilbert

**Affiliations:** 1 Department of Biological and Environmental Sciences, NanoScience Center, University of Jyväskylä, Jyväskylä, Finland; 2 Department of Chemistry, NanoScience Center, University of Jyväskylä, Jyväskylä, Finland; 3 Department of Physics, NanoScience Center, University of Jyväskylä, Jyväskylä, Finland; 4 Faculty of Science and Nanoscience & Nanotechnology, Graduate Program, King Mongkut’s University of Technology Thonburi, Bangkok, Thailand; 5 Te?ted Ltd, Mattilaniemi 6-8, Jyväskylä, Finland

**Keywords:** Catalysis, Graphene, Chemical synthesis

## Abstract

The present study evaluates the role of graphene oxide’s (GO’s) peroxidase-like and inherent/carbocatalytic properties in oxidising silver nitrate (AgNO_3_) to create graphene nanocomposites with silver nanoparticles (GO/Ag nanocomposite). Activation of peroxidase-like catalytic function of GO required hydrogen peroxide (H_2_O_2_) and ammonia (NH_3_) in pH 4.0 disodium hydrogen phosphate (Na_2_HPO_4_). Carbocatalytic abilities of GO were triggered in pH 4.0 deionised distilled water (ddH_2_O). Transmission electron microscope (TEM), scanning electron microscope (SEM), cyclic voltammetry (CV) and UV-Vis spectroscopy aided in qualitatively and quantitatively assessing GO/Ag nanocomposites. TEM and SEM analysis demonstrated the successful use of GO’s peroxidase-like and carbocatalytic properties to produce GO/Ag nanocomposite. UV-Vis analysis indicated a higher yield in optical density values for GO/Ag nanocomposites created using GO’s carbocatalytic ability rather than its peroxidase-like counterpart. Additionally, CV demonstrated that GO/Ag nanocomposite fabricated here is a product of an irreversible electrochemical reaction. Our study outcomes show new opportunities for GO as a standalone catalyst in biosensing. We demonstrate a sustainable approach to obtain graphene nanocomposites exclusive of harmful chemicals or physical methods.

## Introduction

Remarkable optical, thermal, mechanical, and electrical properties of graphene have captivated the imaginations of scientists worldwide^1–4^. Graphene is an ideal composite counterpart to create flexible electronics^5,6^, design batteries with enhanced storage capability^7^, prevent steel corrosion^8^, and shield aircraft from heat^9^. Real-world graphene applications have originated across several industries like the Inov-8 running trainers that employ thermal and mechanical qualities of graphene to improve their durability^10^. The famous sports equipment manufacturer, HEAD applied graphene to create stronger tennis rackets with lighter weight distribution^10^. The BAC-Mono formula racing car also uses graphene to boost strength and reduce the mass of its body parts by 20%^11^. Miniaturization of biochemical assays using graphene-based sensors for research and clinical purposes is now a reality with AGILER100^12^.

Utilization of graphene by Inov-8^10^, HEAD^10^, BAC-Mono^11,12^, and AGILER100^12^ is the tip of an iceberg considering graphene’s estimated market size of $680 million by 2020^13^. However, the reported market size for graphene was a mere $12 million in 2013, indicating that sales in the graphene industry are predominantly raw material driven^14^. Graphene’s annual production has peaked at 1200 tonnes per annum compared to 4600 tonnes per year of carbon fibre or carbon nanotubes^14^. Reported growth in the graphene industry may seem uninspiring but demonstrates the need for technological innovations that will increase graphene consumption. A surge in patent and scientific citation trends hint graphene nanocomposites as a promising area for research and application^13^. Unlike graphene, the role of graphene nanocomposites extends beyond improving the existing physiological properties of a host matrix^13^.

Catalytic properties of graphene nanocomposite help generate or store energy, reduce emission from modern automobiles, and remove pollutants from water, soil, and air^13,15^. Currently, research on graphene nanocomposites heavily relies on physically or chemically coupling graphene with metal nanoparticles for utilization in a spectrum of catalytic processes^16–24^. Thus, understanding graphene’s role as a standalone heterogeneous carbon catalyst (i.e., carbocatalyst^25^) to create nanocomposites by modifying inorganic or organic compounds is required. A simple PubMed search using the term “Graphene AND catalysis” will render over 2000 scientific articles, and the term “Graphene AND carbocatalysis” gives about ten results. Hence, less than 1% of research articles have explored graphene’s inherent or carbocatalytic properties.

Density functional theory (DFT) calculations reveal the potential use of metal-free graphene to stabilize ground state molecules through non-covalent π-π interactions^26^. However, pure graphene does not dissolve in organic or inorganic solvents^27^. Thus, researchers have functionalized the graphene surface with epoxy, hydroxyl, or carboxyl groups to obtain readily soluble graphene oxide (GO)^27,28^. Apart from GO’s carbocatalytic applications, the use of the peroxidase-like catalytic feature of GO^29^ is popular among researchers developing biochemical assays or biosensors to transform organic compounds^30^. Briefly, in the presence of hydrogen peroxide, GO can replace the horseradish peroxidase enzyme to reduce 3,3′,5,5′-Tetramethylbenzidine (TMB) from colourless to blue^29^. Surprisingly, GO’s peroxidase-like and carbocatalytic abilities are unexplored with the inorganic elements^26^.

As a consequence, this study has investigated GO’s peroxidase-like and natural or carbocatalytic oxidation capabilities with inorganic compounds to show new opportunities for graphene as a standalone catalyst in biosensing. Specifically, the role of GO in changing silver nitrate to silver nanoparticles (Ag) to create GO/Ag nanocomposite without the use of extreme physical and chemical redox methods is explained here.

## Materials and Methods

### Materials

Highly concentrated GO dissolved in water with over 95% monolayer content was purchased from Graphenea, Spain^29^. Disodium phosphate (Na_2_HPO_4_), silver nitrate (AgNO_3_), hydrogen peroxide (H_2_O_2_), and ammonia (NH_3_) were purchased from Sigma-Aldrich, Finland^29^.

### Synthesis of GO/Ag nanocomposite using GO’s peroxidase-like catalytic properties

GO surroundings were tailored to stimulate its peroxidase-like catalytic properties^29^ and facilitate the oxidation of aqueous AgNO_3_ to Ag nanoparticles. Therefore, GO/Ag nanocomposite synthesis was achieved by changing the concentration of GO in the presence of AgNO_3_ at a constant concentration and vice versa. Firstly, different GO concentrations (5 µg, 10 µg, 20 µg, 40 µg, 80 µg, and 160 µg) were independently introduced along with 100 mM H_2_O_2_, 0.1 mM AgNO_3_, and 40 mM NH_3_ to a final volume of 1 ml in 25 mM Na_2_HPO_4_ buffer at pH 4.0 and 37 °C. Secondly, several AgNO_3_ concentrations (0.4 mM, 0.8 mM, 1.6 mM, 3.2 mM, 6.4 mM, and 12.8 mM) were separately added with 100 mM H_2_O_2_, 40 µg GO, and 40 mM NH_3_ to a final volume of 1 ml in 25 mM Na_2_HPO_4_ buffer at pH 4.0 and 37 °C.

### Synthesis of GO/Ag nanocomposite using GO’s intrinsic or carbocatalytic abilities

The need for Na_2_HPO_4_, H_2_O_2_, and NH_3_ to create GO/Ag nanocomposite was assessed by lowering Na_2_HPO_4_ concentration (25 mM, 10 mM, 5 mM, 2.5 mM, 0.5 mM, and 0 mM) in the presence of 40 µg GO, 3.2 mM AgNO_3_, 100 mM H_2_O_2_, and 40 mM NH_3_ in total 1 ml solution. Particularly, the 0 mM Na_2_HPO_4_ (i.e., ddH_2_O) experiment was performed with 40 µg GO and 3.2 mM AgNO_3_ in the presence and absence of 100 mM H_2_O_2_ and 40 mM NH_3_ at pH 4.0 and 37 °C. Without Na_2_HPO_4_, H_2_O_2_, and NH_3_, GO will depend on its natural abilities^25^ to convert aqueous AgNO_3_ to Ag nanoparticles.

### Experimental setup, characterization, and data analysis

In the case of experimental controls, all reaction components were included except AgNO_3_ to control for GO and vice versa. Thus, experimental controls have been referred to as GO alone or AgNO_3_ alone hereon. The reaction temperature for all experiments was maintained at 37 °C using a heat block, and each component was added at 15 min intervals^29^. The order in which each chemical was added to Na_2_HPO_4_ buffer was GO, H_2_O_2_, AgNO_3_, and NH_3_. Further, UV-VIS spectral analysis (Perkin Elmer Lambda 650) was performed between 300 nm to 700 nm 15 min after adding the last element. Specimens for changing GO and AgNO_3_ were performed in triplicates to measure average standard deviation across all wavelengths from 300 nm to 700 nm.

Following equation was applied to measure the difference in absorbance between GO/Ag nanocomposite to GO or AgNO_3_ alone,

1 $$Nanocomposite-to-control\,percentage=\left(\frac{Nanocomposite-Control}{Nanocomposite}\times 100\right)$$

wherein *“Nanocomposite”* refers to the GO/Ag nanocomposite absorbance at 450 nm and *“Control”* denotes matching GO or AgNO_3_ alone absorbance at 450 nm. The horseradish peroxidase (HRP) enzyme reacts with 3,3’,5,5’- tetramethylbenzidine (TMB) and is detectable at 450 nm that is extensively used for various biochemical applications^31,32^. As GO can mimic peroxidase-like activity^29^, the nanocomposite-to-control percentage measurements were performed at 450 nm. Equation 1 was adapted from Microsoft Office instructions for calculating percentages using the Microsoft Excel. Further, cyclic voltammetry (CV) was performed to study the molecular electrochemistry between GO and AgNO_3_. Following previously described set-up for CV^33^, an in-house CV was created using glassy carbon electrodes as the working and counter electrodes with an Ag/AgCl reference electrode (66-EE009) from Cypress systems. A Keithley electrometer series 2400 was utilized together with a homebuilt LabView measurement program to measure current (mA) between the working and counter electrodes, as well as difference in potential (V) between the working and reference electrodes. To avoid interference from dissolved O_2_, the electrolyte solution was purged with N_2_ gas. In all CV measurements, ±3 V was applied with a step size of 10 mV and scan rate of 38 mVs^−1^.

Visualization studies were conducted to view the nanocomposite structures and aid in elemental analysis. Transmission electron microscopy (TEM) and scanning electron microscopy (SEM) required 5 μl of sample on Formvar/carbon coated 200 mesh copper grids. In the case of TEM, digital micrographs were acquired with Jeol JEM-1400HC (Jeol, Tokyo, Japan) that is equipped with Quemesa (Olympus Soft Imaging Systems, Münster, Germany) bottom mounted CCD-camera. Chemical characterization for GO/Ag nanocomposite was achieved with Zeiss EVO-50XVP SEM that is integrated with Bruker Quantax 400 energy dispersive spectrometer (EDS). Elemental maps from EDS were obtained using Bruker AXS detector 3001 with energy resolution <133 eV (MnKa, 1000 cps). The counts per second values in elemental maps were normalized. Image J (https://imagej.nih.gov/ij/ version 1.51) was utilized for measuring the average size of Ag nanoparticles on GO and adding a scale bar.

All figures were created using Microsoft Excel (https://products.office.com/en-in/ excel version 16.32 (19120802)), Microsoft PowerPoint (https://products.office.com/en-in/powerpoint version 16.16.3 (181015)), QtiPlot (https://www.qtiplot.com/ version 10.9), Adobe Photoshop 2020 (https://www.adobe.com/in/products/photoshop.html version 21.0.2), and Adobe Illustrator 2020 (https://www.adobe.com/in/products/illustrator.html version 24.0.1).

## Results

### Characterization of GO/Ag nanocomposite created using GO’s peroxidase-like catalytic properties

**Figure 1:**
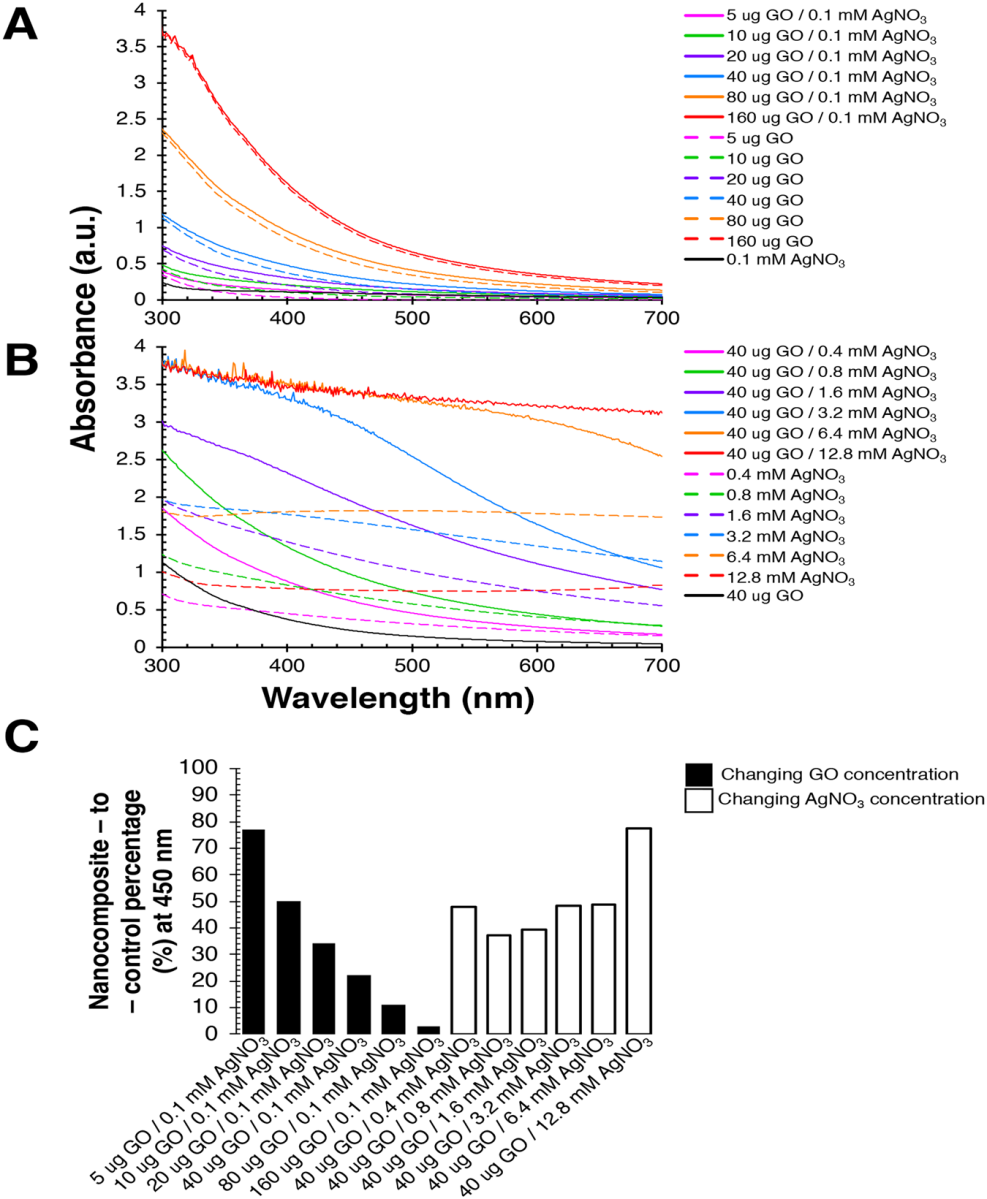
Graphene oxide (GO) utilizes its peroxidase-like catalytic property to oxidize aqueous silver nitrate (AgNO_3_) to silver (Ag) nanoparticles. UV-VIS spectral analysis to evaluate (**A**) the synthesis of GO/Ag nanocomposites at different GO concentrations (5 µg/ml, 10 µg/ml, 20 µg/ml, 40 µg/ml, 80 µg/ml, and 160 µg/ml) in the presence of 0.1 mM AgNO_3_, 25 mM Na_2_HPO_4_, 100 mM H_2_O_2_, and 40 mM NH_3_, (**B**) the synthesis of GO/Ag nanocomposites at different AgNO_3_ concentrations (0.4 mM, 0.8 mM, 1.6 mM, 3.2 mM, 6.4 mM, and 12.8 mM) in the presence of 40 µg/ml GO, 25 mM Na_2_HPO_4_, 100 mM H_2_O_2_, and 40 mM NH_3_, and (**C**) the resulting nanocomposite-to-control percentage at 450 nanometres (nm) between GO and AgNO_3_ together (i.e., experiment) and GO or AgNO_3_ alone (i.e., control) from (**A,B**). All chemical reactions were carried out at 37 °C in pH 4.0 solvents. Absorbance was recorded in arbitrary units (a.u.) from 300 to 700 nm. Solid lines represent absorbance due to interaction between GO and AgNO_3_ together (i.e., experiment) versus dashed lines indicates absorbance of either GO or AgNO_3_ alone (i.e., control). Figure 1 was created using Microsoft Excel (https://products.office.com/en-in/excel version 16.32 (19120802)), Microsoft PowerPoint (https://products.office.com/en-in/powerpoint version 16.16.3 (181015)), and Adobe Photoshop 2020 (https://www.adobe.com/in/products/photoshop.html version 21.0.2).

The reaction between increasing quantities of GO (5 µg/ml, 10 µg/ml, 20 µg/ml, 40 µg/ml, 80 µg/ml, and 160 µg/ml) with 0.1 mM AgNO_3_, 100 mM H_2_O_2_, and 40 mM NH_3_ in 25 mM Na_2_HPO_4_ is linearly proportional to the rise in absorbance values seen between 300 nm to 700 nm (Fig. 1A). Nevertheless, the nanocomposite-to-control percentage (Fig. 1C) at 450 nm between GO/Ag nanocomposite to corresponding GO alone drops from 77% (5 µg GO/0.1 mM AgNO_3_) to 2.93% (160 µg GO/0.1 mM AgNO_3_). The decline in nanocomposite-to-control percentage is observed because varying GO concentrations in the presence of 0.1 mM AgNO_3_ produce spectra that are only GO dominant. However, as rising concentrations of AgNO_3_ (0.4 mM, 0.8 mM, 1.6 mM, 3.2 mM, 6.4 mM, and 12.8 mM) are subjected to 40 µg/ml GO, 100 mM H_2_O_2_, and 40 mM NH_3_ in 25 mM Na_2_HPO_4_, a four-fold increase in absorbance is noted between AgNO_3_ alone and their respective GO/Ag nanocomposites (Fig. 1B).

The nanocomposite-to-control percentage (Fig. 1C) at 450 nm between GO/Ag nanocomposite to matching AgNO_3_ alone rises from 47% (40 µg GO/0.4 mM AgNO_3_) to 77% (40 µg GO/12.8 mM AgNO_3_). Therefore, varying AgNO_3_ amounts in the presence of 40 µg/ml GO yields spectra that are GO/Ag nanocomposite dominant (Fig. 1B). Additionally, Fig. 1B reveals that 40 µg/ml GO/12.8 mM AgNO_3_ nanocomposite spectra is the upper limit for detection as the said GO/Ag combination saturates UV-VIS spectroscopy machine’s capability to absorb. Hence, the maximum nanocomposite-to-control percentage between GO/Ag nanocomposite to GO or AgNO_3_ alone can be either observed at low GO quantities (5 µg GO/0.1 mM AgNO_3_) or at high AgNO_3_ concentration (40 µg GO/12.8 mM AgNO_3_) as depicted in Fig. 1C. Overall, the absorbance values between 300 nm to 700 nm for all GO/Ag nanocomposites created by varying GO and AgNO_3_ concentrations (Fig. 1A,B) were replicable when performed in triplicates with an average standard deviation of 0.008 a.u. and 0.039 a.u., respectively (Fig. S1).

**Figure 2:**
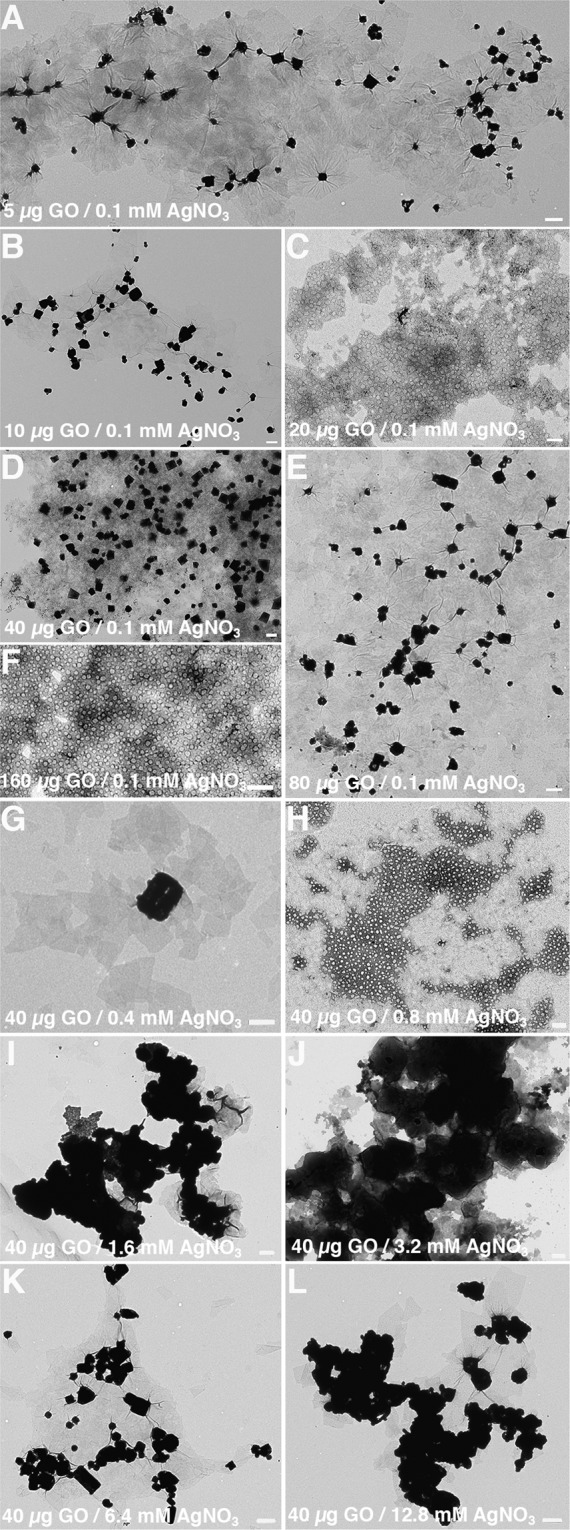
Nucleation of silver (Ag) nanoparticles occurs exclusively on the graphene oxide (GO) surface. Transmission electron microscope (TEM) analysis to evaluate the synthesis of GO/Ag nanocomposites at different GO and AgNO_3_ concentrations. Various GO concentrations include (**A**) 5 µg/ml GO, (**B**) 10 µg/ml GO, (**C**) 20 µg/ml GO, (**D**) 40 µg/ml GO, (**E**) 80 µg/ml GO, and (**F**) 160 µg/ml GO in the presence of 0.1 mM AgNO_3_, 25 mM Na_2_HPO_4_, 100 mM H_2_O_2_, and 40 mM NH_3_. Similarly, several concentrations for AgNO_3_ include (**G**) 0.4 mM AgNO_3_, (**H**) 0.8 mM AgNO_3_, (**I**) 1.6 mM AgNO_3_, (**J**) 3.2 mM AgNO_3_, (**K**) 6.4 mM AgNO_3_, and (**L**) 12.8 mM AgNO_3_ in the presence of 40 µg/ml GO, 25 mM Na_2_HPO_4_, 100 mM H_2_O_2_, and 40 mM NH_3_. All chemical reactions were carried out at 37 °C in pH 4.0 solvents. Bar 500 nm. Figure 2 was compiled with the help of Image J (https://imagej.nih.gov/ij/ version 1.51) and Adobe Photoshop 2020 (https://www.adobe.com/in/products/photoshop.html version 21.0.2).

Electron micrograph images of GO (160 µg/ml) or AgNO_3_ (12.8 mM) alone (Fig. S2) were visually compared with images of GO/Ag nanocomposites (Fig. 2). The black dots or cloud-like deposits on GO sheets in Fig. 2 are Ag nanoparticles in comparison to the clean GO sheets in Fig. S2A. Silver nanoparticles were observed exclusively on GO sheets indicating that GO plays an imperative role in oxidizing aqueous AgNO_3_ and act as a substrate on which the Ag nanoparticles can nucleate (Fig. 2). Although changing GO concentrations in the presence of 0.1 mM AgNO_3_ produce spectra that are GO dominant (Fig. 1A), silver nanoparticles can still get deposited on GO sheets (Fig. 2A–F). Nucleation of Ag nanoparticles on GO sheet was not restricted and the resulting anisotropic nanoparticles measured larger than 500 nm (Fig. 2A–F). On increasing the amounts of AgNO_3_, the continuous growth of Ag nanoparticles into colloidal cloud-like particles on GO sheets was noted (Fig. 2G–L).

### Characterization of GO/Ag nanocomposite produced using GO’s intrinsic or carbocatalytic abilities

GO can utilize its peroxidase-like catalytic property to oxidize aqueous AgNO_3_ to Ag nanoparticles (Figs. 1A,B, and 2). Nonetheless, the nanocomposite-to-control percentage at 450 nm between GO/Ag nanocomposite to GO or AgNO_3_ alone is limited to 77% (Fig. 1C). Growing the difference in absorbance between GO/Ag nanocomposite and GO or AgNO_3_ alone at 450 nm should elevate the nanocomposite-to-control percentage (Eq. 1). To assess the reagents that produce high noise, absorbance values of 25 mM Na_2_HPO_4_, 100 mM H_2_O_2_, and 3.2 mM AgNO_3_ were collected at 450 nm in several combinations (Fig. S3). Individually, 25 mM Na_2_HPO_4_, 100 mM H_2_O_2_, and 3.2 mM AgNO_3_ did not exhibit noteworthy absorbance. In contrast, a twelve-fold increase in absorbance was observed when 3.2 mM AgNO_3_ was introduced to 25 mM Na_2_HPO_4_ (Fig. S3). An additional three-fold rise in absorbance was noted when 100 mM H_2_O_2_ was subjected to 3.2 mM AgNO_3_ in 25 mM Na_2_HPO_4_.

**Figure 3:**
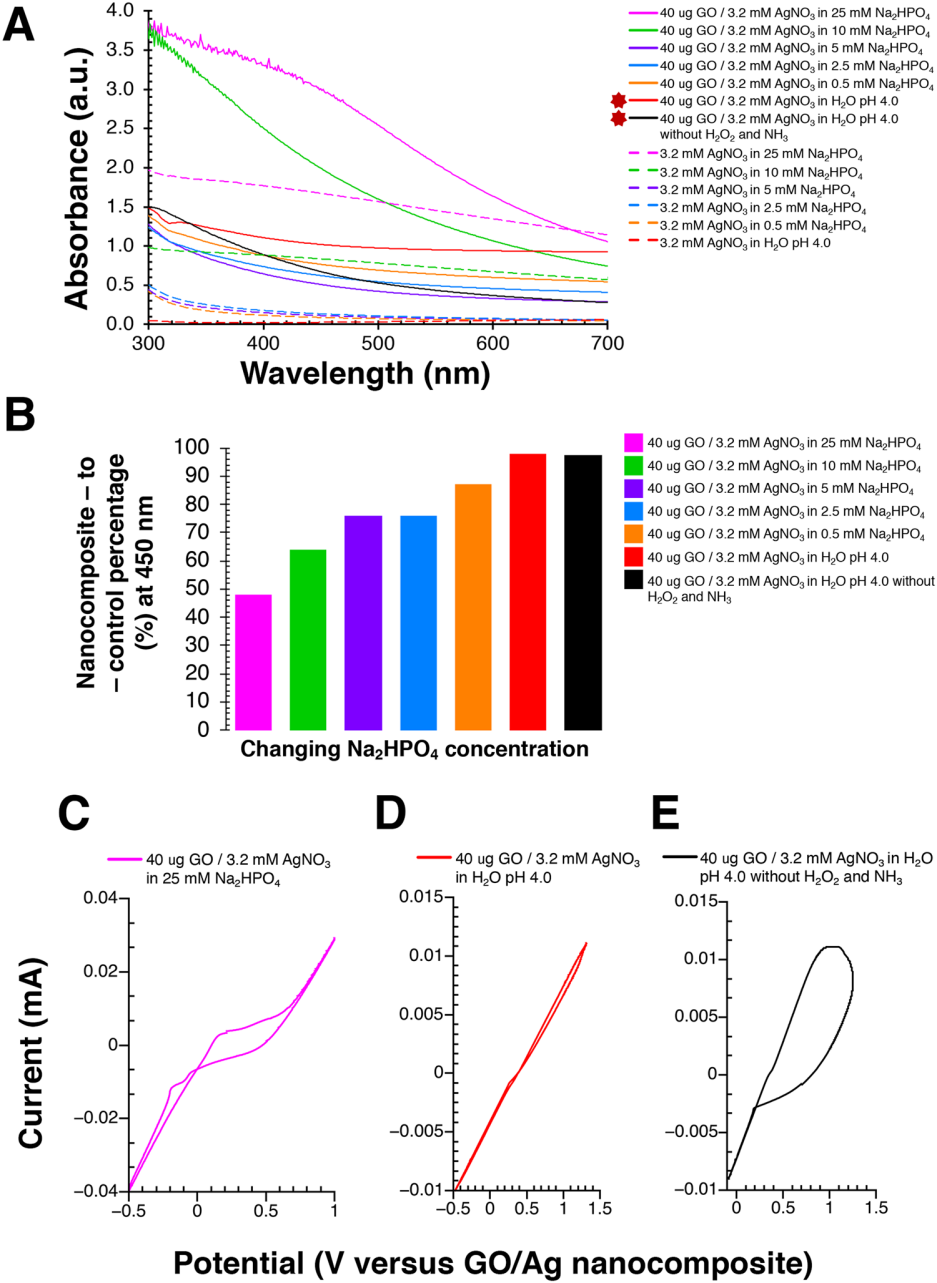
Graphene oxide (GO)/silver (Ag) nanocomposite results from an irreversible electrochemical reaction that demonstrates the largest difference in absorbance without di-sodium hydrogen phosphate (Na_2_HPO_4_), hydrogen peroxide (H_2_O_2_), and ammonia (NH_3_). UV-VIS spectral analysis to evaluate (**A**) the synthesis of GO/Ag nanocomposites at different Na_2_HPO_4_ concentrations (25 mM, 10 mM, 5 mM, 2.5 mM, 0.5 mM, and 0 mM) in the presence of 40 µg/ml GO, 3.2 mM AgNO_3_, 100 mM H_2_O_2_, and 40 mM NH_3_. Specifically, the 0 mM Na_2_HPO_4_ (i.e., H_2_O) experiment was performed with 40 µg/ml GO and 3.2 mM AgNO_3_ in the presence and absence of 100 mM H_2_O_2_, and 40 mM NH_3_ and has been indicated with dark red stars, and (**B**) the resulting nanocomposite-to-control percentage at 450 nanometres (nm) between GO and AgNO_3_ together (i.e., experiment) and GO or AgNO_3_ alone (i.e., control) from Fig. 1A,B. Further, cyclic voltammograms for 40 µg/ml GO and 3.2 mM AgNO_3_ with (**C**) 25 mM Na_2_HPO_4_, 100 mM H_2_O_2_, and 40 mM NH_3_, (**D**) H_2_O, 100 mM H_2_O_2_, and 40 mM NH_3_, and (**E**) H_2_O without 100 mM H_2_O_2_ and 40 mM NH_3_ indicates electrochemical irreversibility. All chemical reactions were carried out at 37 °C in pH 4.0 solvents. Absorbance was recorded in arbitrary units (a.u.) from 300 to 700 nm. Solid lines represent absorbance due to interaction between GO and AgNO_3_ together (i.e., experiment) versus dashed lines indicates absorbance of either GO or AgNO_3_ alone (i.e., control). In all CV measurements, ±3 V was applied with a step size of 10 mV and scan rate of 38 mVs^−1^. Image J (https://imagej.nih.gov/ij/ version 1.51) was utilized for measuring the average size of Ag nanoparticles on GO and adding a scale bar. Figure 3 was assembled using Microsoft Excel (https://products.office.com/en-in/excel version 16.32 (19120802)), Microsoft PowerPoint (https://products.office.com/en-in/powerpoint version 16.16.3 (181015)), QtiPlot (https://www.qtiplot.com/ version 10.9), Adobe Photoshop 2020 (https://www.adobe.com/in/products/photoshop.html version 21.0.2), and Adobe Illustrator 2020 (https://www.adobe.com/in/products/illustrator.html version 24.0.1).

Interaction between AgNO_3_ and Na_2_HPO_4_ leads to high absorbance background that is further exacerbated by the addition of H_2_O_2_ (Fig. S3). Lowering Na_2_HPO_4_ concentration (25 mM, 10 mM, 5 mM, 2.5 mM, and 0.5 mM) in the presence of 3.2 mM AgNO_3_, 100 mM H_2_O_2_, and 40 mM NH_3_ gradually decreases the background produced by AgNO_3_ controls (Fig. 3A). Similarly, the decline in Na_2_HPO_4_ concentration in the presence of 40 µg/ml GO with 3.2 mM AgNO_3_, 100 mM H_2_O_2_, and 40 mM NH_3_ steadily reduces GO/Ag nanocomposite absorbance spectra (Fig. 3A). The nanocomposite-to-control percentage measured at 450 nm between AgNO_3_ alone and matching GO/Ag nanocomposite climbs from 48% for 25 mM Na_2_HPO_4_ to 87% for 0.5 mM Na_2_HPO_4_ (Fig. 3B). Interestingly, GO/Ag nanocomposite’s nanocomposite-to-control percentage grows to 97% (Fig. 3B) in the absence of Na_2_HPO_4_ and H_2_O_2_ (Fig. 3A).

### Electrochemical behaviour and elemental analysis of GO/Ag nanocomposite

The electrochemical measurements between GO and AgNO_3_ in Na_2_HPO_4_ or H_2_O yielded cyclic voltammograms with a large capacitive current contribution due to the working electrode having a large surface area. A redox response is most visible in the presence of Na_2_HPO_4_ with an oxidation peak at 0.15 V and a reduction peak at −0.1 V (Fig. 3C). GO and AgNO_3_ in H_2_O however displays no visible redox response and GO/Ag nanocomposite seems to be fully stable (Fig. 3D). When H_2_O_2_ and NH_3_ is removed, the CV response shows a broad oxidation curve that suggests a sluggish electron exchange between GO and AgNO_3_ (Fig. 3E). In contrast, no discernible oxidation (anodic) or reduction (cathodic) curves were observed when GO or AgNO_3_ alone were dissolved in H_2_O (Fig. S4).

**Figure 4:**
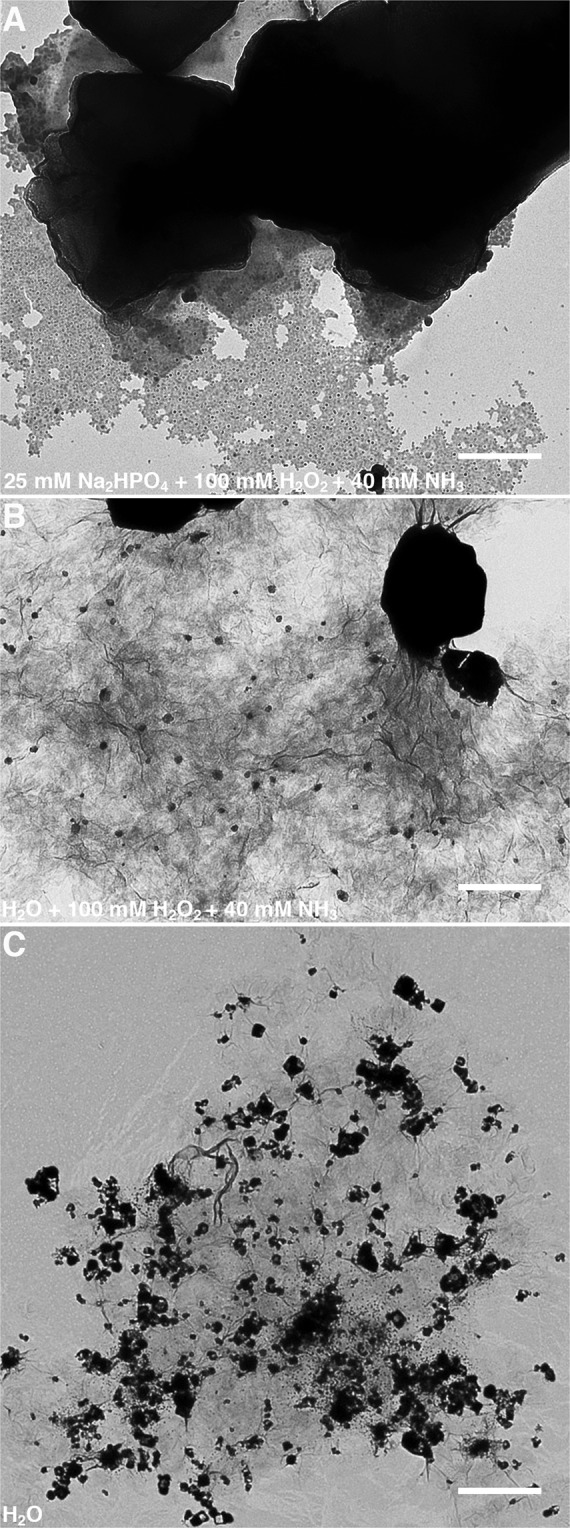
Graphene oxide (GO) utilizes its intrinsic or carbocatalytic ability to oxidize aqueous silver nitrate (AgNO_3_) to silver (Ag) nanoparticles in the absence of di-sodium hydrogen phosphate (Na_2_HPO_4_), hydrogen peroxide (H_2_O_2_), and ammonia (NH_3_). Transmission electron microscope (TEM) analysis to evaluate the facile synthesis of GO/Ag nanocomposites in (**A**) 25 mM Na_2_HPO_4_ with 100 mM H_2_O_2_ and 40 mM NH_3_, (**B**) H_2_O with 100 mM H_2_O_2_ and 40 mM NH_3_, (**C**) H_2_O without 100 mM H_2_O_2_ and 40 mM NH_3_. All chemical reactions were carried out at 37 °C in pH 4.0 solvents using 40 µg/ml GO with 3.2 mM AgNO_3_. Bar 500 nm. Figure 4 was compiled with the help of Image J (https://imagej.nih.gov/ij/ version 1.51) and Adobe Photoshop 2020 (https://www.adobe.com/in/products/photoshop.html version 21.0.2).

**Figure 5:**
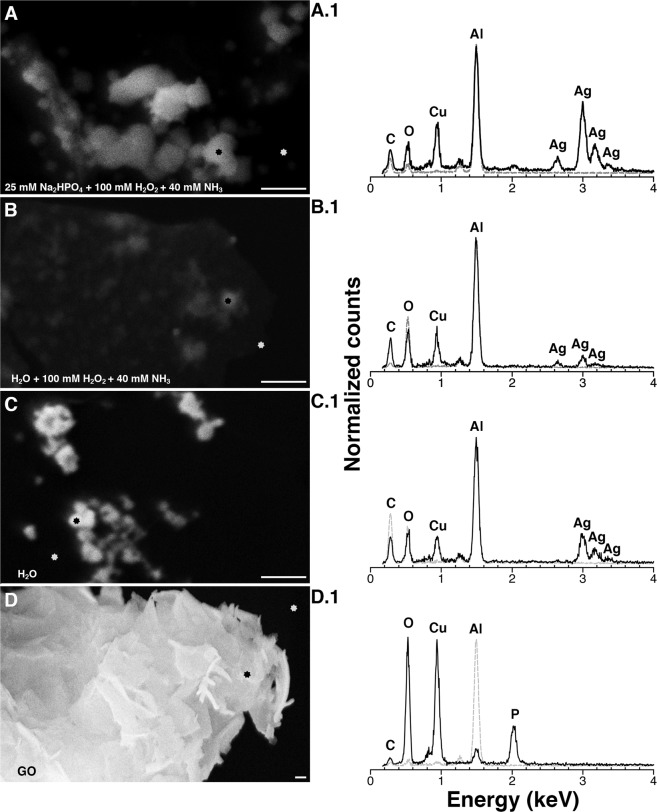
Scanning electron microscopy (SEM) and energy dispersive spectrometer (EDS) provide empirical evidence for graphene oxide (GO)/silver (Ag) nanocomposite fabrication. SEM micrographs indicates the synthesis of GO/Ag nanocomposites in (**A**) 25 mM Na_2_HPO_4_ with 100 mM H_2_O_2_ and 40 mM NH_3_, (**B**) H_2_O with 100 mM H_2_O_2_ and 40 mM NH_3_, (**C**) H_2_O without 100 mM H_2_O_2_ and 40 mM NH_3_, and (**D**) GO alone. The black and grey stars in Fig. 5A–D indicate the place for EDS measurements that directly corresponds with the black and dashed grey lines in elemental maps for GO/Ag nanocomposites in (A.1) 25 mM Na_2_HPO_4_ with 100 mM H_2_O_2_ and 40 mM NH_3_, (B.1) H_2_O with 100 mM H_2_O_2_ and 40 mM NH_3_, (C.1) H_2_O without 100 mM H_2_O_2_ and 40 mM NH_3_, and (D.1) GO alone. All chemical reactions were carried out at 37 °C in pH 4.0 solvents using 40 µg/ml GO with 3.2 mM AgNO_3_. Bar 500 nm. Figure 5 was created using Microsoft PowerPoint (https://products.office.com/en-in/powerpoint version 16.16.3 (181015)), Adobe Photoshop 2020 (https://www.adobe.com/in/products/photoshop.html version 21.0.2), and Adobe Illustrator 2020 (https://www.adobe.com/in/products/illustrator.html version 24.0.1).

The result of electrochemical behaviour between GO and AgNO_3_ can be seen in the electron micrographs presented in Fig. 4. TEM images visibly indicate the deposition of silver nanoparticles on GO sheets in the absence of Na_2_HPO_4_ (Fig. 4B) and H_2_O_2_ (Fig. 4C) that is comparable to GO/Ag nanocomposite created in 25 mM Na_2_HPO_4_ (Figs. 2 and 4A). Additionally, the presence of silver nanoparticles on GO sheets was confirmed with SEM and EDS (Fig. 5). Here the GO/Ag nanocomposite appears bright compared to the darker background of formvar. Mapping of elements was performed either on (black star) or around (grey star) the GO/Ag nanocomposite (Fig. 5A–C). Similarly, elemental maps include a black or grey line to correspond with said coloured EDS measurement stars (Fig. 5A.1–C.1). Elemental mapping on GO/Ag nanocomposite (black star and line) consistently demonstrated peaks for Carbon (C at 0.28 keV), Oxygen (O at 0.53 keV), Copper (Cu at 0.93 keV), Aluminium (Al at 1.49 keV), and Silver (Ag at 2.99 keV, 3.17 keV, and 3.33 keV). The EDS measurements around GO/Ag nanocomposite (grey star and line) did not yield any Cu or Ag peaks, while the C and O peaks are consistent with the underlying formvar. Figure 5D,D.1 indicate that GO is the source for Cu, as also seen in GO/Ag nanocomposite elemental maps (Fig. 5A.1–C.1). Further, Al was seen in all EDS measurements due to the sample holder beneath the formvar copper grid.

**Figure 6:**
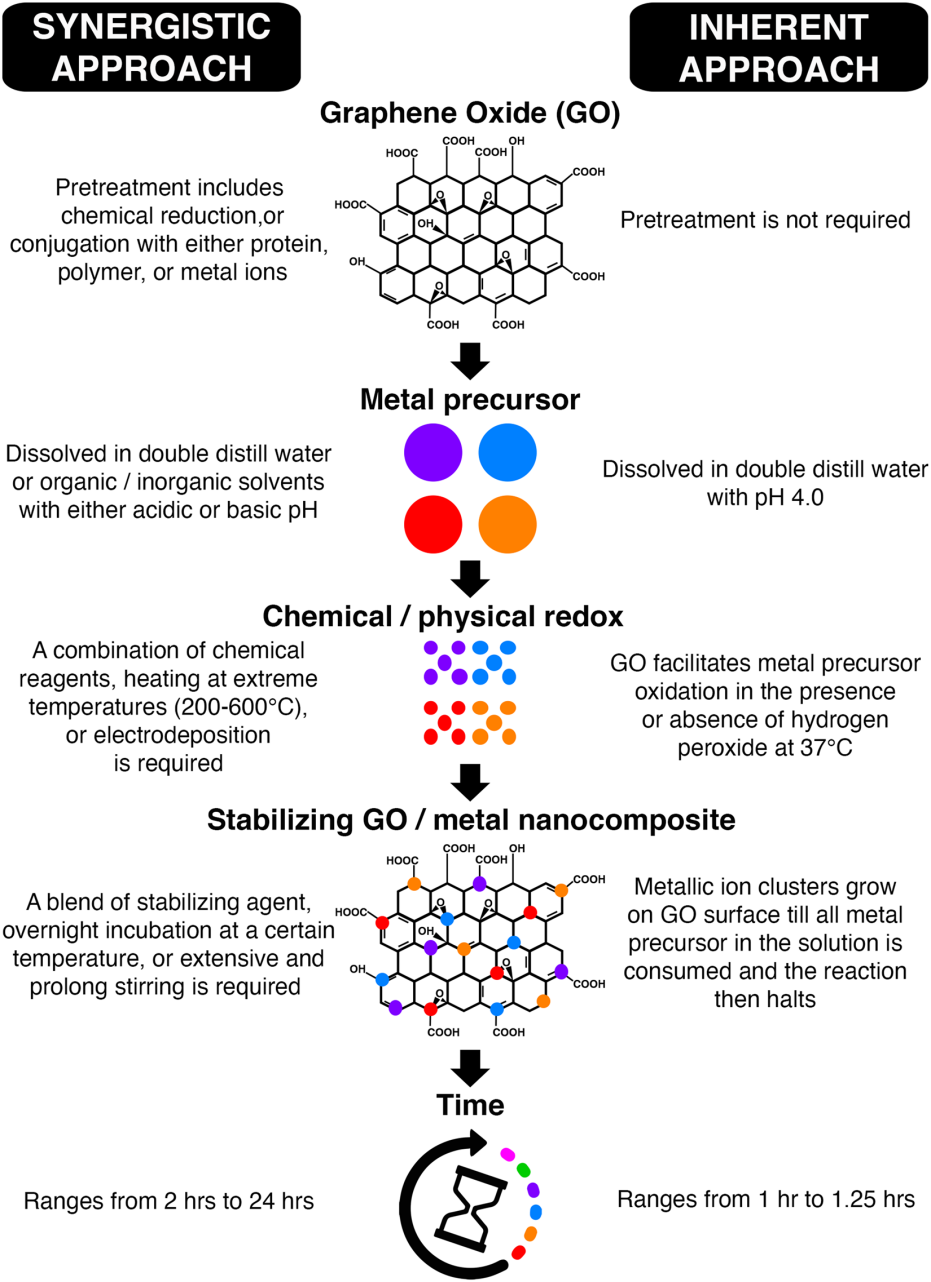
Graphene oxide (GO)/metal nanocomposites can be achieved without the use of harmful chemicals and extreme physical reduction methods. Schematic comparing key requirements to generate GO/metal nanocomposites following reported methodologies (old approach) versus current technique (new approach). Figure 6 was created using Microsoft PowerPoint (https://products.office.com/en-in/powerpoint version 16.16.3 (181015)), Adobe Photoshop 2020 (https://www.adobe.com/in/products/photoshop.html version 21.0.2), and Adobe Illustrator 2020 (https://www.adobe.com/in/products/illustrator.html version 24.0.1).

**Figure 7:**
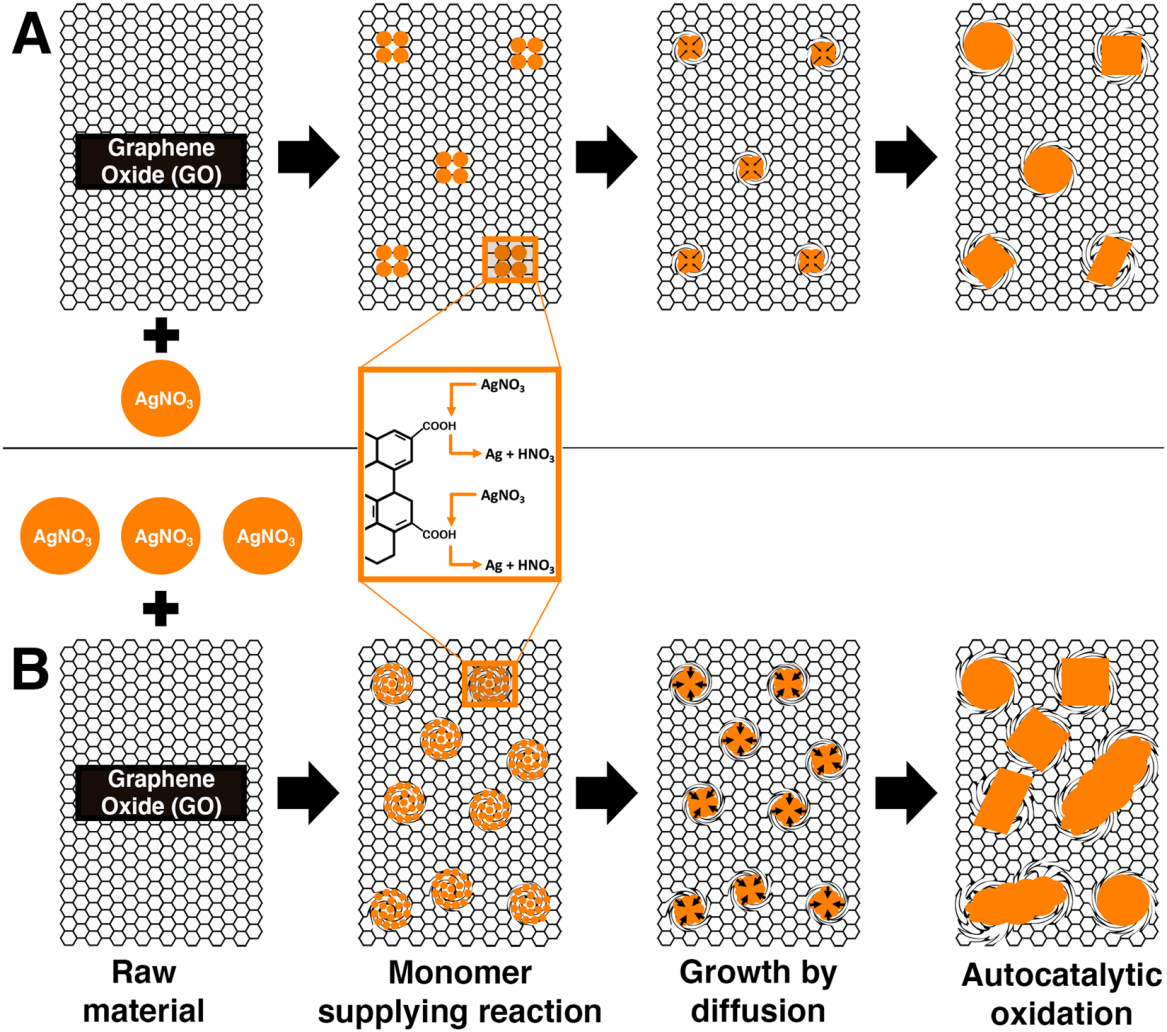
Silver (Ag) nanoparticle growth on graphene oxide (GO) is due to coalescence. Schematic illustration of surface chemistry and the mechanism postulated to be involved in GO/Ag nanocomposite synthesis due to coalescence with (**A**) small, and (**B**) large amounts of AgNO_3_. Figure 7 was created using Microsoft PowerPoint (https://products.office.com/en-in/powerpoint version 16.16.3 (181015)), Adobe Photoshop 2020 (https://www.adobe.com/in/products/photoshop.html version 21.0.2), and Adobe Illustrator 2020 (https://www.adobe.com/in/products/illustrator.html version 24.0.1).

Figure 6 illustrates a comparison between the synergistic and inherent catalytic approaches for creating graphene nanocomposites. Furthermore, Fig. 7 proposes a mechanism to understand the surface chemistry involved in oxidizing AgNO_3_ to Ag nanoparticles.

## Discussion

We demonstrate the use of GO’s peroxidase-like^29^ and inherent or carbocatalytic properties^25^ to oxidize inorganic compounds such as AgNO_3_. The Na_2_HPO_4_ buffer with H_2_O_2_ activates the peroxidase-like catalytic property of GO that converts AgNO_3_ to Ag nanoparticles (Figs. 1 and 2). The nanocomposite-to-control percentage for resulting GO/Ag nanocomposite is a maximum of 77% (Fig. 1C) because the interaction between Na_2_HPO_4_ and AgNO_3_ produces high noise (Fig. S3). In contrast, GO/Ag nanocomposite created in deionized distilled water (pH 4.0) without H_2_O_2_ yields 97% in nanocomposite-to-control difference (Fig. 3B). In the absence of Na_2_HPO_4_ and H_2_O_2_, GO’s intrinsic ability facilitates the transformation of AgNO_3_ to Ag nanoparticles (Figs. 3–5). Large aromatic basal planes of GO offer a high surface area containing epoxy, hydroxyl, and carboxyl functional groups that contribute to GO’s inbuilt catalytic properties^25^. Our findings indicate that GO alone can oxidize AgNO_3_ and also act as a substrate for Ag nanoparticles to generate GO/Ag nanocomposites.

The GO/Ag nanocomposite created in this study using only natural catalytic properties of GO is simple, resource-efficient, and swift compared to old techniques (Fig. 6). Traditionally, synthesis of GO nanocomposites with metal nanoparticles as shown in Fig. 6 includes pre-treatment of GO, organic/inorganic solvents to dissolve the metal precursor, and chemical/physical methods to reduce and stabilize the final product^16–24^. GO pre-treatment may involve adsorption or electrodeposition of metal ions^16^, conjugation with antibodies^17^, PEG^19^, and more^18,23^. Further, solvents of metal precursors such as AgNO_3_^16^, chloroplatinic acid (H_2_PTCL_6_)^21^, and cobalt (II) acetate (Co(CH3COO)_2_)^22^ include deionized distilled water^19^, ethanol^22^, 1% BSA^17^, citrate buffer^16^, or sulphuric acid^23^. Ultimately, blending of GO with metal nanoparticles demands the presence of chemical agents like hydroquinone^16,17^, hydrazine hydrate^20^, sodium borohydride (NaBH_4_)^24^, or ammonium hydroxide (NH_4_OH)^22^ to reduce and stabilize the metal clusters on GO surface. Alternatively, extreme physical methods such as autoclave^15,22^, annealing or heating at 60 °C to 600 °C^19,20,23^ have been reported to create metal nanoparticles over GO.

Catalytic properties of GO are either synergistic or inherent following the literature^15^ and our research findings (Figs. 4–6). A synergistic approach advocates the integration of GO with other catalytic components such as silver^16^, palladium^20^, platinum^21^, or cobalt^22^ nanoparticles using pre-treated GO^16–20,23^, chemical agents^16,17,20–22,24^, or heating^19,20,22,23^. As a result, GO in synergy with metal nanoparticles participates in photocatalysis^24^, photodegradation^23^, bactericidal activity^16^, Suzuki-Miyaura coupling reaction^20^, oxygen evolution reaction^22^, and methanol^21^ or oxygen oxidation reaction^22^. In contrast, the oxidation of AgNO_3_ to Ag nanoparticles in this study is influenced by the functional group composition, structure, and morphology of GO (Figs. 3–5) that signifies its inherent catalytic properties^25^. Application of GO’s intrinsic catalytic abilities is limited to either hydration or oxidation of various organic compounds, including alcohols, alkynes, and alkenes^34^. Additionally, the use of heteroatom doped GO in oxygen reduction reactions is considered as a natural use of GO’s catalytic abilities^34–36^. However, attaching heteroatoms like sulphur^36^ and iodine^35^ to GO also comprises several pre-treatment processes as vigorous ultrasonication, and annealing at 500 °C to 1100 °C in an argon atmosphere^35,36^.

The GO/Ag nanocomposite fabricated here is a product of an electrochemical irreversible reaction (Fig. 3C,D). Kinetics that may govern the irreversible nucleation, growth, and attachment of Ag nanoparticles on GO (Fig. 7) can be described in three steps (1) monomer supplying reaction, (2) growth by diffusion and (3) autocatalytic oxidation^37,38^. Same three steps also explain the development of gold nanoparticles in a classical Turkevich method wherein citrate and squaric or ascorbic acid are used as reducing agents^37–43^. The monomer supplying reaction (Fig. 7) is imperative for making Ag monomers/ions and clusters^37,38^. Primary contact between AgNO_3_ and functional groups of GO may initiate the creation of many Ag monomers over GO. Coalescence follows the creation of Ag monomers due to the low aggregation barrier to form first clusters or Ag seed particles (Fig. 7). Lower or higher aggregation barrier is essentially activation energy that either promotes coalescence among monomers or inhibits aggregation between large nanoparticles, respectively^37,38,40^. In the next step, newly formed Ag monomers may diffuse onto the existing seed particles to facilitate the growth phase of Ag nanoparticles over GO^38,40^. Diffusion rate for the growth of Ag nanoparticles may strongly depend on the amount of Ag monomers that are supplied to standing clusters (Fig. 7). Lastly, autocatalytic oxidation comprises the constant reiteration of steps one and two till remaining AgNO_3_ is consumed to obtain the final Ag nanoparticles^37,38^.

Unlike the Turkevich method^37–42^, the size, shape, density, and distribution of Ag nanoparticles on GO is not homogenous in our study. Nonetheless, heterogenous GO/Ag nanocomposites have produced replicable results when characterized using UV-VIS spectroscopy (Fig. S1). The growth by diffusion step is crucial for narrowing polydispersity among gold nanoparticles that are produced in suspension using the Turkevich method^37–42^. In contrast, nucleation and growth of Ag nanoparticles are restricted by GO’s finite sheet size (Figs. 2, S2, 5) and island-like arbitrary distribution^44,45^ of active sites (i.e., epoxy, hydroxyl, and carboxyl functional groups). Also, the size and density of Ag nanoparticles on GO is determined by the starting quantity of AgNO_3_ (Fig. 7). Small amounts of raw AgNO_3_ are readily converted to Ag nanoparticles that are distinguishable (Fig. 7A). However, larger quantities of AgNO_3_ are transformed to Ag cloud-like nanoparticles that prominently cover the GO sheets (Fig. 7B). Cloud-like nanoparticles may result due to sizeable AgNO_3_ residual that is continuously oxidized to Ag monomers and diffused onto the existing Ag nanoparticles (i.e., autocatalytic oxidation) over a finite size of GO sheet (Fig. 7B).

Among various inorganic compounds, applications of silver nanoparticles in the biomedical field is widespread and ever-increasing due to its low-cost, abundance, and fascinating properties^46,47^. As a consequence, silver nitrate was utilized in this study to embody GO’s peroxidase-like and natural or carbocatalytic oxidation capabilities with inorganic compounds. However, as Ag is monovalent, more research is required to understand the limits of GO’s peroxidase-like or intrinsic catalytic properties using divalent, trivalent, and tetravalent inorganic elements^48^. The intrinsic and peroxidase-like catalytic properties of graphene shown here may be seen in its allotropes like carbon nanotubes^49–52^ and fullerenes^53^ coupled with epoxy, hydroxyl, and carboxyl groups. Also, in future experiments, radical scavenger study and electron spin resonance technique can help understand the influence of free radicals, O_2_, and OH groups (i.e., resulting from decomposed H_2_O_2_) on the fabrication of carbon nanocomposites.

What plastic was for the 20th century, graphene is to the 21st century. The World Bank and the United Nations have adopted several sustainable development goals to reduce the risk that plastic poses to our environment and public health^54^. Currently, the lack of standardized health and safety guidelines to assess the toxic influence of unique graphene-based technologies on human health and the environment is staggering^14^. In Europe, the registration, evaluation, authorization, and restriction of chemicals (REACH) regulation (EC 1907/2006) by the European Agency for Safety and Health at Work instructs wellbeing at organizations that manufacture or import harmful chemicals^55^. Nonetheless, the REACH regulation only applies to establishments dealing with one tonne or more graphene per year^14^. In 2017, the International Organization for Standards (ISO) published ISO/TS 80004:13:2017^56^ that only clarifies technical pre-requisites for a material to be deemed as graphene or otherwise.

Given the inevitable mass application of graphene and missing guidelines to prevent public health hazards and environmental risks, the research community may become the frontline in consciously assessing the carbon footprint of their innovations. In the spirit of new opportunities for graphene in catalysis like biosensing, we show that using GO’s standalone catalytic abilities will reduce the widespread dependence on harmful chemicals and extreme physical methods to obtain graphene nanocomposites.

## Data Availability

All available data in present article has been illustrated through Figs. 1 to 7 and Figures S1 to S4.

## Supplementary Information

Supplementary Information.
