# Supplementary material for: Preparation of graphene nanocomposites from aqueous silver nitrate using graphene oxide’s peroxidase-like and carbocatalytic properties
Source: Sci Rep. 2020 Mar 20;10:5126. doi: 10.1038/s41598-020-61929-9 (PMC7083964; doi:10.1038/s41598-020-61929-9)
Supplement: Supplementary file 1 — Supplementary Information. [file 41598_2020_61929_MOESM1_ESM.docx]

**Supplementary Information**

**Preparation of graphene nanocomposites from aqueous silver nitrate using graphene oxide’s peroxidase-like and carbocatalytic properties**

Kunal Garg^1,5^, Petri Papponen^1^**^§^**, Andreas Johansson^2,3^**^§^**, Nitipon Puttaraksa^4^, Leona Gilbert* ^1,5^

^1^Department of Biological and Environmental Sciences, NanoScience Center, University of Jyväskylä, Jyväskylä, Finland.

^2^Department of Chemistry, NanoScience Center, University of Jyväskylä, Jyväskylä, Finland.

^3^Department of Physics, NanoScience Center, University of Jyväskylä, Jyväskylä, Finland.

^4^Faculty of Science and Nanoscience & Nanotechnology, Graduate Program, King Mongkut’s University of Technology Thonburi, Bangkok, Thailand.

^5^Te?ted Ltd, Mattilaniemi 6-8, Jyväskylä, Finland.

*Corresponding author

Email: [leona.k.gilbert@jyu.fi](mailto:leona.k.gilbert@jyu.fi)

**^§^** These authors contributed equally to this work.

**
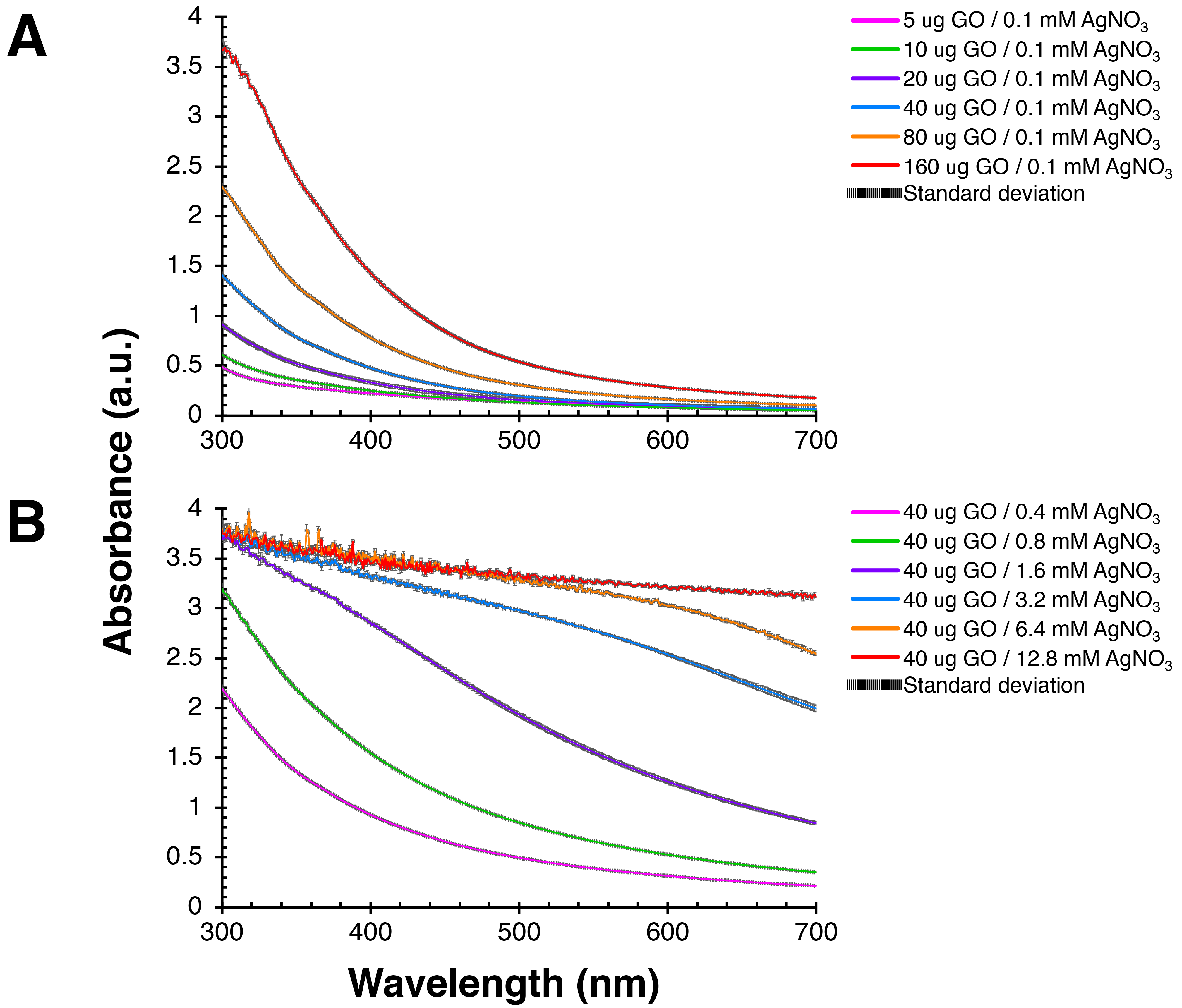
**

**Figure S1. Absorbance values for graphene oxide (GO) / silver (Ag) nanocomposite experiments are highly reproducible.**  UV-VIS spectral evaluation of GO / Ag nanocomposites in triplicates at different (A) GO concentrations (5 µg/ml, 10 µg/ml, 20 µg/ml, 40 µg/ml, 80 µg/ml, and 160 µg/ml) in the presence of 0.1 mM AgNO_3_, 25 mM Na_2_HPO_4_, 100 mM H_2_O_2_, and 40 mM NH_3_, (B) AgNO_3_ concentrations (0.4 mM, 0.8 mM, 1.6 mM, 3.2 mM, 6.4 mM, and 12.8 mM) in the presence of 40 µg/ml GO, 25 mM Na_2_HPO_4_, 100 mM H_2_O_2_, and 40 mM NH_3_. All chemical reactions were carried out at 37°C in pH 4.0 solvents. Absorbance was recorded in arbitrary units (a.u.) from 300 to 700 nanometres (nm). Figure S1 was created using Microsoft Excel (<https://products.office.com/en-in/excel> version 16.32 (19120802)), Microsoft PowerPoint (<https://products.office.com/en-in/powerpoint> version 16.16.3 (181015)), and Adobe Photoshop 2020 (<https://www.adobe.com/in/products/photoshop.html> version 21.0.2).


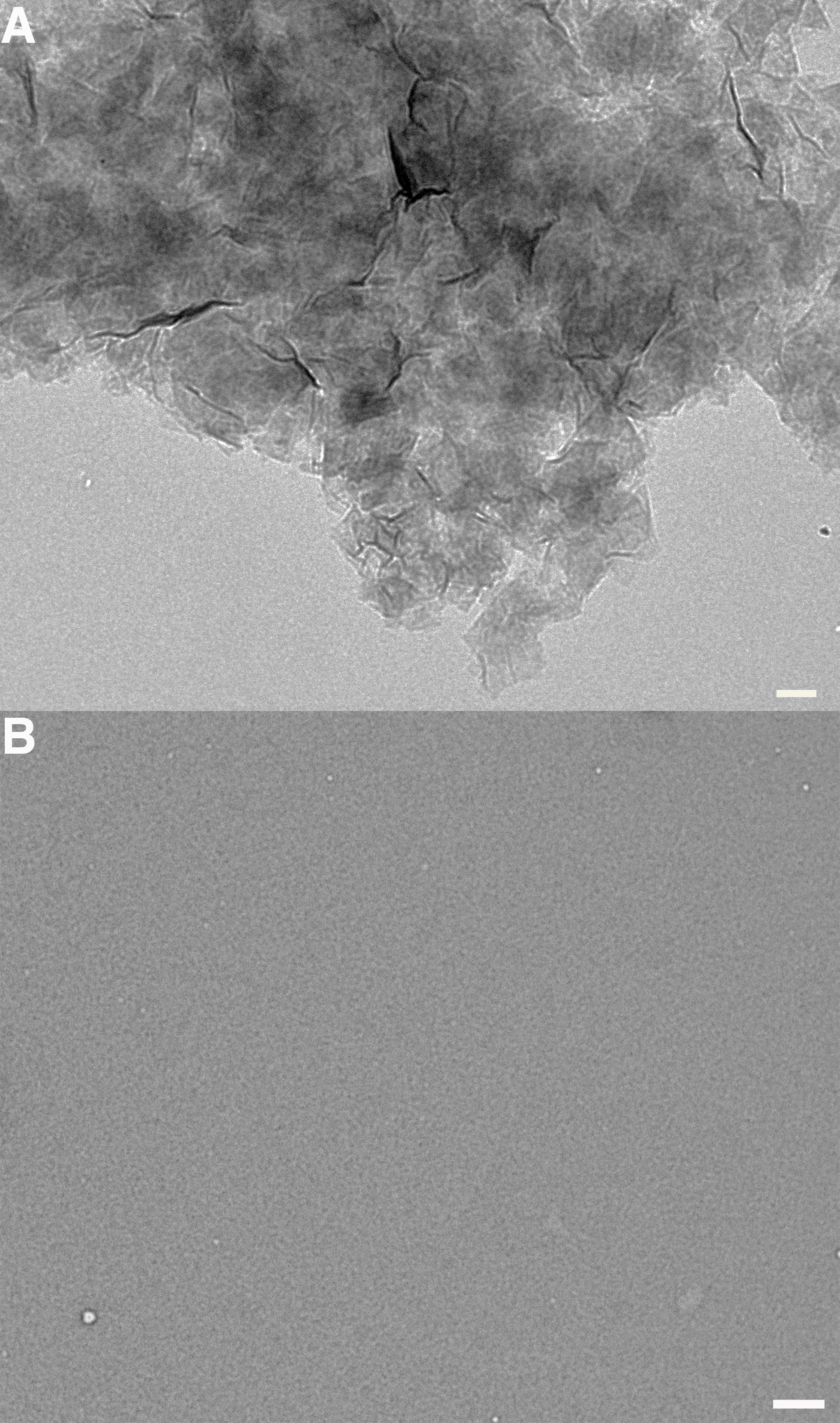


**Figure S2.** **Aqueous** **silver nitrate (AgNO_3_) cannot be oxidized to silver (Ag) nanoparticles in the absence of graphene oxide (GO).** Transmission electron microscope (TEM) visualization of (A) 160 µg/ml GO, and (B) 12.8 mM AgNO_3_ controls in the presence of 25 mM Na_2_HPO_4_, 100 mM H_2_O_2_, and 40 mM NH_3_. Figure S2 was compiled with the help of Image J (<https://imagej.nih.gov/ij/> version 1.51) and Adobe Photoshop 2020 (<https://www.adobe.com/in/products/photoshop.html> version 21.0.2).


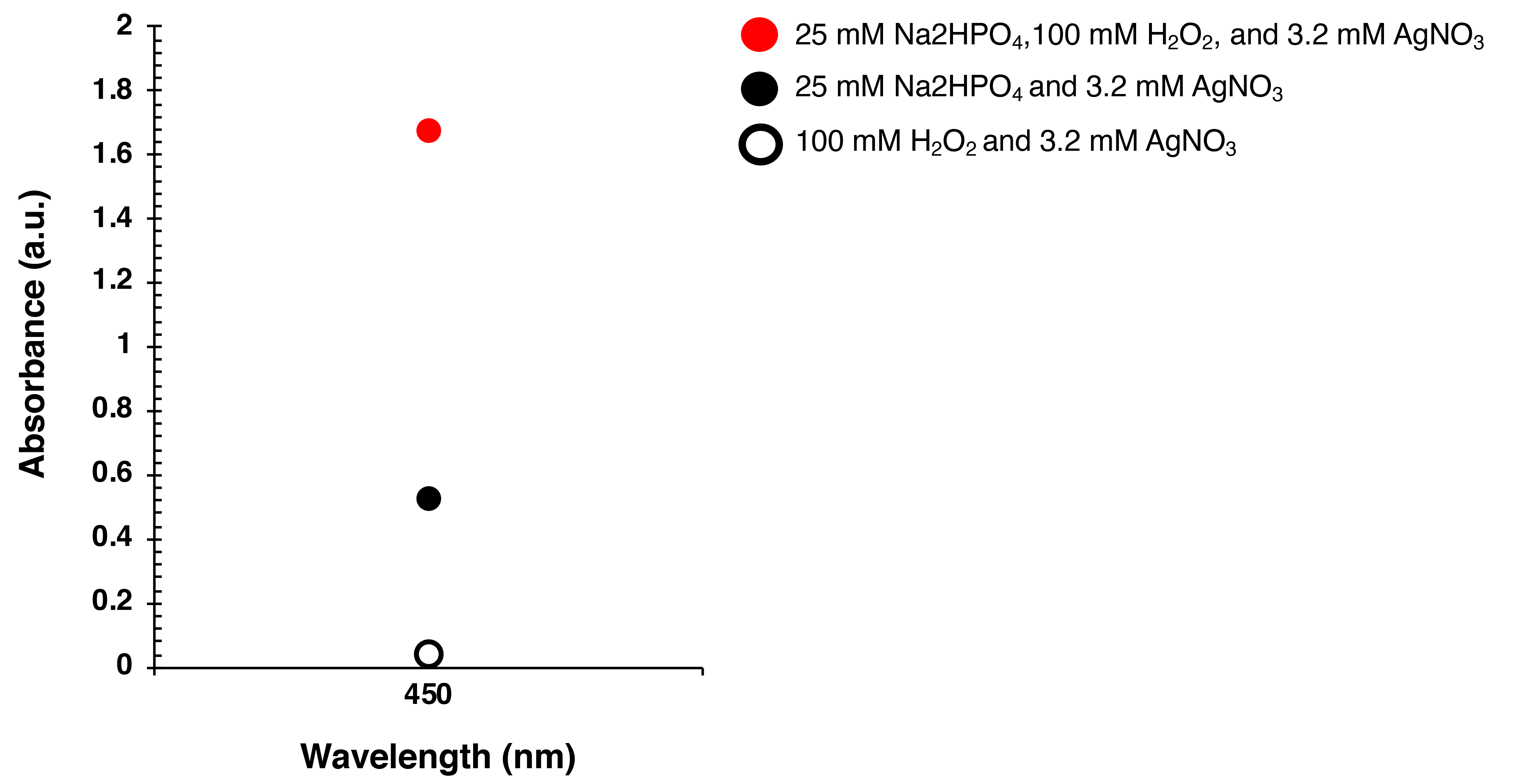


**Figure S3. Di-sodium hydrogen phosphate (Na_2_HPO_4_) interacts with silver nitrate (AgNO_3_) to produce high background absorbance in the absence of graphene oxide (GO).** The absorbance values of 25 mM Na_2_HPO_4_, 100 mM H_2_O_2_, and 3.2 mM AgNO_3_ were considered independently and in combination at 450 nm to evaluate the key components contributing to large background absorbance. Figure S3 was assembled using Microsoft Excel (<https://products.office.com/en-in/excel> version 16.32 (19120802)), and Adobe Photoshop 2020 (<https://www.adobe.com/in/products/photoshop.html> version 21.0.2).


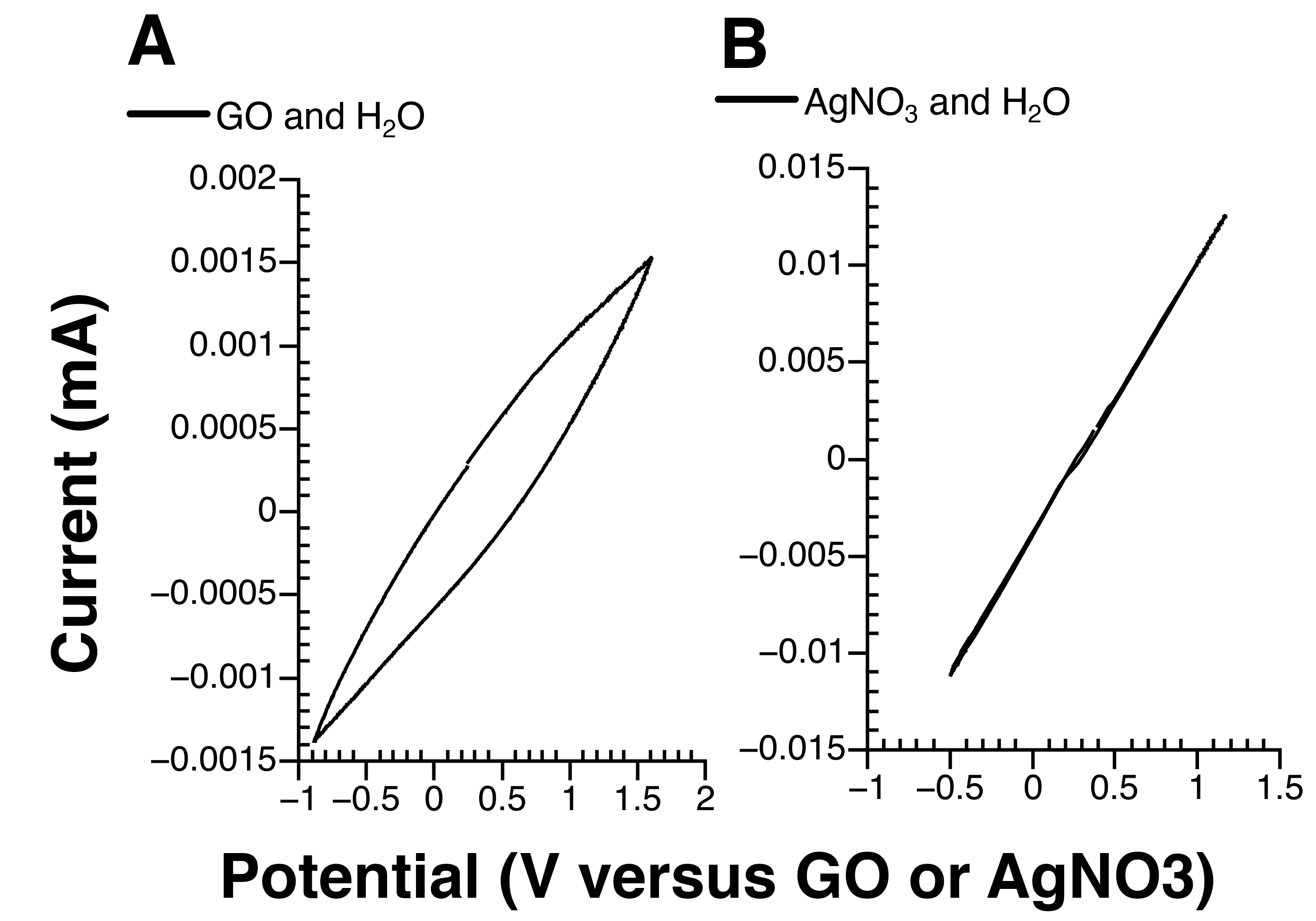


**Figure S4. No oxidation or reduction electrochemical behaviour is observed when graphene oxide (GO) or silver nitrate (AgNO_3_) alone are dissolved in water H_2_O.** Cyclic voltammograms for (A) 40 µg/ml GO and (B) 3.2 mM AgNO_3_ alone dissolved in H_2_O. In all CV measurements, ±3 V was applied with a step size of 10 mV and scan rate of 38 mVs^-1^. Figure S4 was assembled using QtiPlot (<https://www.qtiplot.com/> version 10.9), Adobe Photoshop 2020 (<https://www.adobe.com/in/products/photoshop.html> version 21.0.2), and Adobe Illustrator 2020 (<https://www.adobe.com/in/products/illustrator.html> version 24.0.1).
